# Immaturity of the Oculomotor Saccade and Vergence Interaction in Dyslexic Children: Evidence from a Reading and Visual Search Study

**DOI:** 10.1371/journal.pone.0033458

**Published:** 2012-03-16

**Authors:** Maria Pia Bucci, Naziha Nassibi, Christophe-Loic Gerard, Emmanuel Bui-Quoc, Magali Seassau

**Affiliations:** 1 Laboratoire de Psychologie et Neuropsychologie Cognitives, FRE 3292 CNRS - Université Paris Descartes, Boulogne Billancourt Cedex, France; 2 Service de Psychopathologie de l'enfant et de l'adolescent, Hôpital Robert Debré, Paris, France; 3 Service d'Ophtalmologie, Hôpital Robert Debré, Paris, France; 4 e(ye)BRAI, Ivry-sur-Seine, France; California Pacific Medicial Center Research Institute, United States of America

## Abstract

Studies comparing binocular eye movements during reading and visual search in dyslexic children are, at our knowledge, inexistent. In the present study we examined ocular motor characteristics in dyslexic children *versus* two groups of non dyslexic children with chronological/reading age-matched. Binocular eye movements were recorded by an infrared system (mobileEBT®, e(ye)BRAIN) in twelve dyslexic children (mean age 11 years old) and a group of chronological age-matched (N = 9) and reading age-matched (N = 10) non dyslexic children. Two visual tasks were used: text reading and visual search. Independently of the task, the ocular motor behavior in dyslexic children is similar to those reported in reading age-matched non dyslexic children: many and longer fixations as well as poor quality of binocular coordination during and after the saccades. In contrast, chronological age-matched non dyslexic children showed a small number of fixations and short duration of fixations in reading task with respect to visual search task; furthermore their saccades were well yoked in both tasks. The atypical eye movement's patterns observed in dyslexic children suggest a deficiency in the visual attentional processing as well as an immaturity of the ocular motor saccade and vergence systems interaction.

## Introduction

The term “dyslexia” has its origins in 1887 when an ophthalmologist described reading difficulty; after more than a century of research it is still unclear what dyslexia exactly is [1].

Reading is a higher cognitive process depending on multiple processes: sensorial perception, eye movements, linguistic and semantic capacities. Deficits in one or more of such mechanisms could be at the origin of dyslexia. Despite intensive research on such issues the origin of dyslexia is still debated, and many theories have been proposed [2].

For instance, a large amount of data has shown that eye movements during reading are abnormal in dyslexia and this has been reported in different languages. Pavlidis [3] was the first to show a high number of regressive saccades and unstable fixation in dyslexic population; Rayner [4] reported frequent saccades of smaller amplitude as well as longer duration fixation in dyslexic children; similarly, De Luca et al. [5] observed frequent fixations with longer duration in Italian dyslexic children and Hutzler & Wimmer [6], also showed a high number of fixations and short duration in dyslexic children. Furthermore, in Chinese dyslexic children, Li et al. [7] reported abnormal eye movements in picture searching, slow and more fixations and frequent saccades of small amplitude. Recently, Trauzettel-Klosinski et al. [8] reported in German dyslexic children slower reading speed and high number of saccades and regressions; similar findings have been also reported in Greek dyslexic children by Hatzidaki et al. [9].

Taken together all these findings suggest that the abnormal eye movement performance observed in dyslexic children could be due to poor ability and strategy of visual information processing. Recently Jainta & Kapoula [10] reported in dyslexics poor binocular coordination of saccades during reading as well as in non reading task (while exploring a painting), while other studies on dyslexic children comparing reading and non reading tasks found ocular motor deficits in the reading task only and made the hypothesis of a deficiency of a higher psycholinguistic level of processing [11],[12].

The presence of a poor visual system in dyslexics has been suggested since many years [13] who first reported dysfunction at the level of the magnocellular system in dyslexics. Following this work, many studies confirmed this hypothesis showing in dyslexic children poor binocular coordination during prolonged fixations [14], visual confusion during reading [15] and poor eye alignment during fixation after the saccade [16]. Iles et al. [17] also reported an impairment in visual search performance in a group of dyslexic adults with a motion coherence deficit confirming and extending the magnocellular hypothesis of dyslexia. Despite these results, recent research did not share the hypothesis of poor visual system, and the existence of a deficiency in the magnocellular system in dyslexia is still under debate [18],[19]. Maybe as suggested by Vidyasagar & Pammer [20] deficit in dyslexia could be localized anywhere along the dorsal stream.

Furthermore, one should also mention that apart from the visual perceptual deficiencies, visual attentional processes are involved in reading and they could be responsible for altered eye movements' performance in dyslexic population. In this line of thinking, Bosse et al. [21] reported that some dyslexic children have a reduced visual attentional window size leading to a limitation in the number of letters which can be processed in parallel. A consequence of such a disorder is that dyslexics will make shorter saccades and frequent fixations with respect to non dyslexic children not only during reading task but also during visual search [22]. A recent fMRI study of this group [23] provided evidence on the role of parietal regions, particularly the left superior parietal area, in the visual attentional span and its deficiency in dyslexics.

In the present study, we wonder to assess the quality of ocular motor coordination in reading and visual search tasks. Studies comparing binocular eye movements during reading and visual search in dyslexic children are, at our knowledge, inexistent.

Ocular motor coordination in dyslexic children will be compared with that observed in a group of non dyslexic children of similar chronological age, and also in a group of non dyslexic children of similar reading age. Indeed, according to our previous studies exploring binocular coordination in normal [24] as well as in children with vergence abnormalities [25],[26] we made the hypothesis that the poor quality of binocular coordination of saccades could be related to immaturity of normal ocular motor learning mechanism responsible of a fine control between the saccades and the vergence command. Such learning mechanisms could grow up with visual experiences during daylife leading to an improvement in binocular coordination during childhood [27]. Our driven hypothesis is that the saccade and vergence interaction in dyslexic children is immature with respect to their chronological age. To test this hypothesis we explored whether ocular motor performance of dyslexic children was more similar to those of a group of younger children (reading age matched) rather than to those of a group of chronological age matched children.

## Materials and Methods

### Subjects

Twelve dyslexic children participated in the study. Dyslexic children were recruited from the pediatric hospital where they were referred for a complete evaluation of their dyslexia state with an extensive examination including neurological/psychological and phonological capabilities. For each child the time of reading a text, its comprehension, and the capacity of reading word/pseudowords were evaluated by using the L2MA battery [28]. This is the standard test developed by the Centre de Psychologie appliquée de Paris, often used in France and already employed in our previous studies for selecting dyslexic population [26],[29]. Inclusion criteria were: scores of this test beyond 2 standard deviations; a normal mean intelligence quotient (IQ, evaluated with WISC-IV; between 80 and 115). The mean age of the dyslexic children was 11±0.6 years, the mean IQ was 100±7 and the mean reading age was 8.8±1 years. A carefully selected chronological age-matched group (mean age: 11±0.9 years) of 9 non-dyslexic children and reading age-matched group of 10 non-dyslexic children (age: 8.3±0.9 years) were selected. Both groups of non dyslexic children had to satisfy the following criteria: no known neurological or psychiatric abnormalities, no history of reading difficulty, no visual impairment or difficulty with near vision. Also, their reading capabilities were in normal range. Both the similitude test of the WISC IV assessing the verbal capability, and the matrix test of the WISC IV assessing the logic capability were performed. Normal range for both tests is 10±3 (Wechsler intelligence scale for children—fourth edition, 2004). The selected reading age-matched group was normal for verbal (11.78±0.8) and for logic (9.97±0.6) capabilities. The selected age-matched group was also normal (10.36±0.4 for verbal and 11.89±0.5 for logic).

Both non-dyslexic and dyslexic children underwent an ophthalmologic examination of their visual sensorial and motor function (mean values showed in Table 1). All children had normal binocular vision (mean value of 55 s of arc or better), which was evaluated with the TNO random dot test. Visual acuity was normal (≥20/20) for all children, dyslexic as well as non dyslexic. The near point of convergence was normal for all three groups of children tested (mean value of 2 cm). Heterophoria at near distance (i.e. latent deviation of one eye when the other eye is covered, using the cover-uncover test) was normal for all three groups of children tested (≤exophoria of 3.5 prism D). Moreover, an evaluation of vergence fusion capability using prisms and Maddox rod was done at near distance. The divergence and convergence amplitudes were significantly different in the dyslexic group with respect to the other two groups of non dyslexic children. ANOVA showed a significant group effect for the divergence and convergence amplitudes (respectively, F_(2,28)_ = 4.74, p<0.01 and F_(2,28)_ = 4.47, p<0.02). The LSD test showed that the dyslexic group had significantly smaller values of divergence with respect to the reading age-matched group (p<0.005) while they had significantly smaller values of convergence amplitudes with respect to the two groups of non dyslexic children (younger, p<0.02 and older, p<0.01).

**Table 1:** Clinical characteristic of the three groups of children examined (dyslexic, D 10–12; non dyslexic children reading age matched, ND 7–9; and non dyslexic children chronological age matched, ND 10–12).

	TNO	NPC	Heterophoria	Divergence	Convergence
**D 10–12**	55	2	Exo 3.5	10	24
**ND 7–9**	48	2	Exo 2	16*	38*
**ND 10–12**	35	2	Exo 2	12	39*

Mean values of: binocular vision (Stereoacuity test, TNO measured in seconds of arc; near point of convergence, NPC measured in cm; Heterophoria at near distance measured in prism diopters; Exo = exophoria; Vergence fusional amplitudes (divergence and convergence) at near distance measured in prism diopters. Asterisks indicate that value is significantly different with respect to the group of dyslexic children (p≤0.01).

In summary, orthoptic evaluation showed a tendency of poor divergence and convergence amplitude in dyslexic children.

The investigation adhered to the principles of the Declaration of Helsinki and was approved by our Institutional Human Experimentation Committee (CPP Ile de France I, Hôpital Hotel-Dieu). Written consent was obtained from the children's parents after an explanation of the experimental procedure.

### Ocular motor paradigms

Stimuli were presented on a PC screen of 22″, its resolution was 1920×1080 and the refresh rate was 60 Hz. Note that even if it is well known that intermittent illumination could affect saccade accuracy and visual assessment [30], such refresh rate was sufficient to assure a normal saccade performance.

The reading and visual search tasks are similar to those used by Prado et al. [22] and are described below.

**Figure 1:**
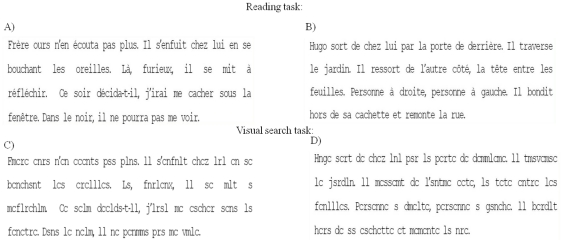
Oculomotor paradigms. Reading (A and B) and visual search (C and D) task respectively used for children with reading age of 7–9 and 10–12 years, respectively.

**Reading:** A text of four lines taken from a book for children. The paragraph contained 40 words and 174 characters. The text was 29° wide and 6.4° high; mean character width was 0.5° and the text was written in black “courier” font on a white background. Text was different for the two different ages of children examined. Figure 1 A and B shows the text presented to children with reading age of 7–9 years (extract from ‘*Jojo Lapin fait des farces*’, Gnid Bulton, Hachette) and that presented to children with reading age of 10–12 years (extracted from ‘*Bagarres à l'école*’, Marc Cantin et Eric Gasté, Castro Cadet). Children were asked to read the text silently.

**Visual search:** The same text presented in the reading task was used for such task but vowels were replaced by consonants (see Figure 1 C and D). Children were asked to count the number of ‘r’ occurring in the text.

In both tasks stimuli were presented without time limitation. The recording of each task stopped when child raised one finger.

### Eye movement recordings

Eye movements were recorded with the Mobile Eyebrain Tracker (Mobile EBT**®**, e(ye)BRAIN, www.eye-brain.com), an eye-tracking device CE marked for medical purpose. The Mobile EBT® benefits from cameras that capture the movements of each eye independently. Recording frequency was set up to 300 Hz. The precision of this system is typically 0.5° and in controlled condition 0.25° (see www.eye-brain.com, for more details). There is no obstruction of the visual field with the recording system.

### Procedure

Children were seated in a chair in a dark room, with the head stabilized by a forehead and chin support; viewing was binocular; the viewing distance was 58 cm. Calibration was done at the beginning of eye movements recordings. The best calibration could be an haploscopic arrangement. However, it should be noted that binocular vision was normal for all children tested (see stereoacuity scores in Table 1), suggesting that they were fixating targets with both eyes. A previous study from Bucci et al. [31] comparing normal and strabismic children confirmed that in the absence of strabismus either type of calibration (under monocular or binocular viewing) was valid.

During the calibration procedure, children were asked to fixate a grid of 13 points (diameter 0.5 deg) mapping the screen. Each calibration point required a fixation of 250 ms to be validated. A polynomial function with five parameters was used to fit the calibration data and to determine the visual angles. After the calibration procedure, the reading or visual search tasks were presented to the child. Duration of the each task was kept short (lasting a couple of minutes) allowing an accurate evaluation of eye movement recordings.

### Data analysis

Calibration factors for each eye were determined from the eye positions during the calibration procedure. The software MeyeAnalysis (provided with the eye tracker, e(ye)BRAIN, www.eye-brain.com, France) was used to extract saccadic eye movements from the data. It determines automatically the onset and the end of each saccade by using “built-in saccade detection algorithm”. All detected saccades were verified by the investigator and corrected/discarded if necessary.

For each saccade recorded in the two tasks (reading and visual search) we examined the amplitude of the conjugate [(left eye+right eye)/2], and the disconjugate components (left eye - right eye) during the saccade. The disconjugacy was measured as the change in vergence between the beginning and the end of each saccade [24], [25], [26]. We also examined the disconjugate component of the post-saccadic drift over the period between two saccades. The duration and the number of those fixations were also evaluated.

Statistical analysis was performed by the three-way ANOVAs using the three groups of children (dyslexics and non-dyslexics, chronological and reading age-matched) as inter-subject factor and the two conditions (reading text and visual search) as within subject factor. The effect of a factor is significant when the p-value is below 0.05.

## Results

### Eye movement pattern during reading and visual search

**Figure 2:**
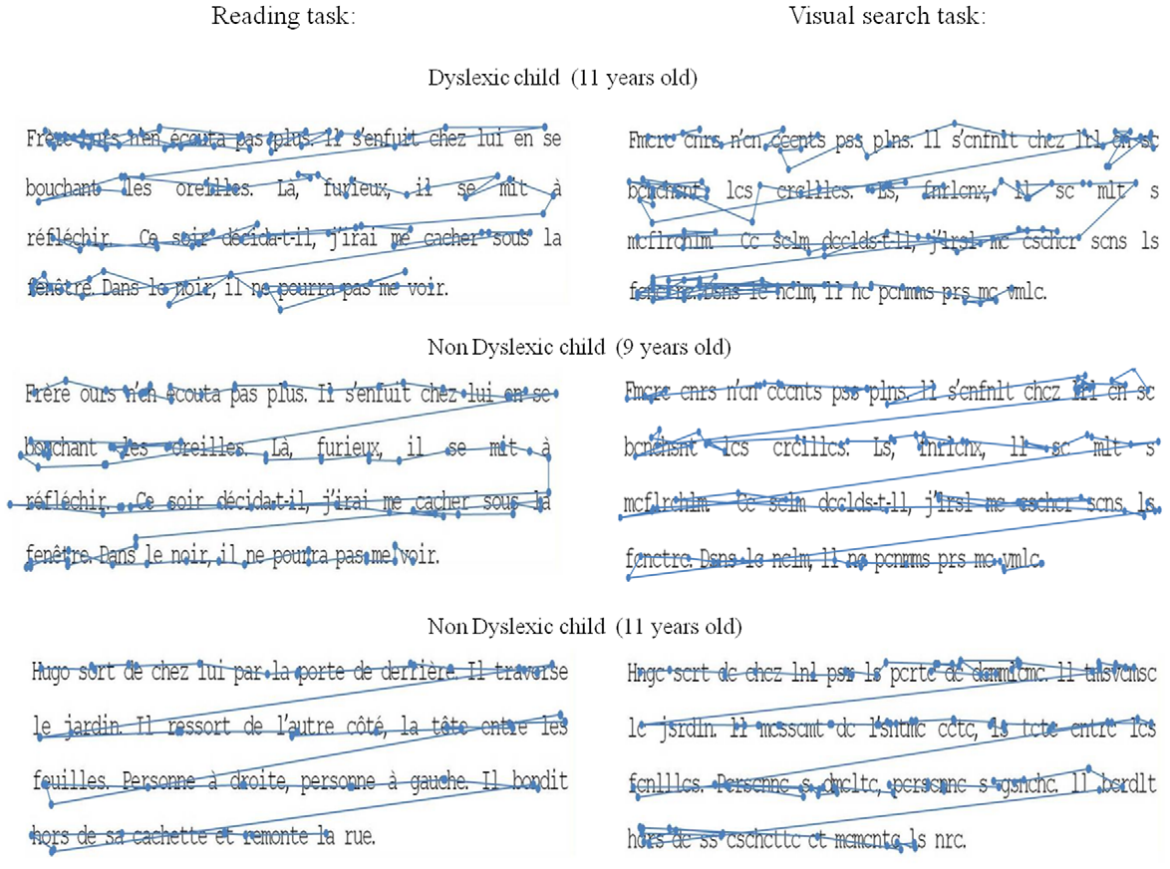
Eye movement pattern during reading and visual search. Number and duration of fixations from dominant right eye the in x- and y-coordinates from a dyslexic child (11 years old) from a non dyslexic child (9 years old) and from a non dyslexic child (11 years old).

Figure 2 shows an example of eye movement patterns from a dyslexic child (11 years old), a non dyslexic child with similar reading age (9 years old) and a non dyslexic child with similar age (11 years old), during the reading task and the visual search task. The dyslexic child showed many fixations independently of the task (reading or visual search); furthermore, he also made frequent backward saccades. The non dyslexic child of 9 years old showed a pattern similar to that of the dyslexic child: many fixations in both tasks. In contrast, the non dyslexic child with similar age (11 years old) showed few fixations in the reading task, suggesting that reading capabilities are working well at that age; while in the visual search task he made many fixations.

In Table 2 the number of fixations assessed during reading and visual search is shown for the three groups of children examined. The ANOVA showed a significant group effect (F_(2,28)_ = 16.64, p<0.0001). Post hoc comparison showed that the number of fixations for the older group of non dyslexic children was significant smaller with respect to that of the dyslexic group (p<0.001) and of the younger group of non dyslexic children (p<0.007).

**Table 2:** Mean number of fixations (± their standard error) in the reading and visual search task for the three groups of children examined (dyslexic, D 10–12; non dyslexic children reading age matched, ND 7–9; and non dyslexic children chronological age matched, ND 10–12).

	Reading	Visual search
**D 10–12**	95±9	100±11
**ND 7–9**	80±6	83±5
**ND 10–12**	36±3	73±6

We found also a significant effect of the task (F_(2,28)_ = 13.24, p<0.001), meaning that the number of fixations was larger in the visual search task with respect to the reading task. Finally, a significant interaction between group and task has been also reported (F_(2,28)_ = 4.05, p<0.03): the older group of non dyslexic children made less fixations during reading than during visual search.

**Figure 3:**
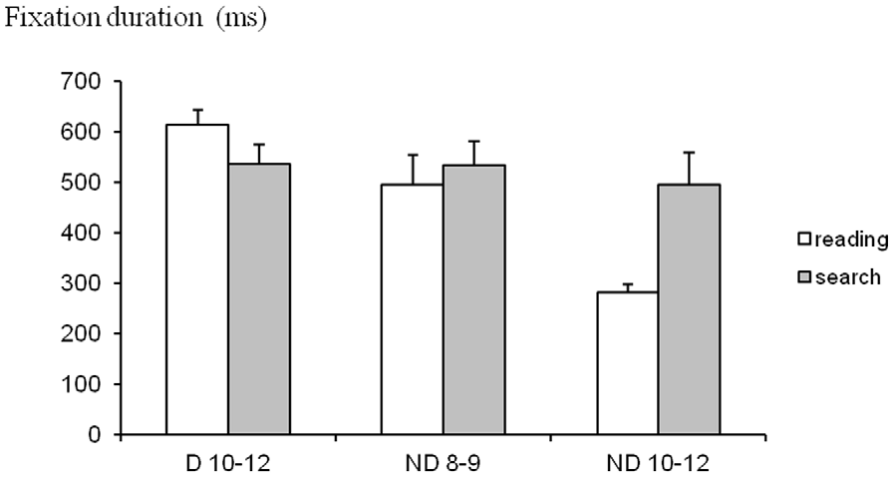
Fixation duration ata. Mean values of fixation duration (in ms) during reading and during visual search for the three groups of children tested. Vertical lines indicate the standard error.

In order to assess more information about fixations, we also measured the average duration of fixations, which is the time period between two saccades (see Figure 3). The ANOVA showed a significant group effect (F_(2,28)_ = 8.40, p<0.001): the duration of fixation of the older group of non dyslexic children was significantly shorter with respect to the dyslexic group of children (p<0.0003) and to the younger group of non dyslexic children (p<0.01).

We found a significant interaction between group and task (F_(2,28)_ = 6.27, p<0.005); more precisely, the older group of non dyslexic children showed shorter duration of fixations in reading task with respect to the visual search task (p<0.04). The ANOVA did not show a significant task effect (F_(2,28)_ = 3.00, p<0.94).

Finally, the performance in the visual search task has been also measured (see Method section) by asking to the child the number of ‘r’ read in the text. Such performance was similar in dyslexic children and in the other two groups of non dyslexic children (younger and older) suggesting that all children accomplished this task in a similar way. The mean value of their performance was 8.9±0.2, 8.7±0.4, 9.1±0.4, respectively in the three groups of children examined.

### Saccade amplitude

**Figure 4:**
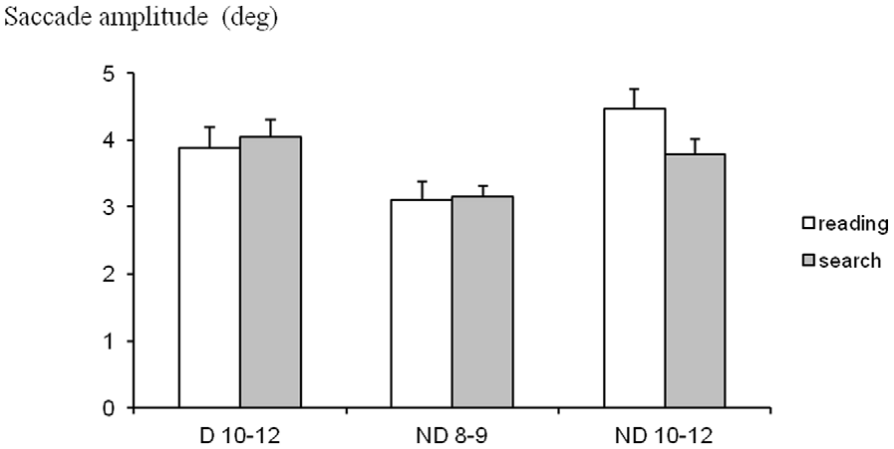
Amplitude of saccades. Mean values of amplitude of saccades (in deg) during reading and during visual search for the three groups of children tested. Vertical lines indicate the standard error.

The absolute mean amplitude of saccades during reading and visual search task for each group of children is shown in Figure 4. The ANOVA showed a significant group effect (F_(2,28)_ = 4.78, p<0.01) and a significant interaction between groups of children and the tasks (F_(2,28)_ = 3.53, p<0.04). Post hoc comparison showed that the amplitude of saccades of the younger group of non dyslexic children was significant smaller with respect to the dyslexic group (p<0.01) and to the older group of non dyslexic children (p<0.008). The amplitude of saccades during reading task for the older group of non dyslexic children was significantly larger with respect to the other groups of children in both reading and visual search tasks (p<0.001). The ANOVA failed to show any significant task effect (F_(2,28)_ = 1.41, p<0.24).

### Disconjugacy during and after the saccades

Given that saccade disconjugacy depends on the saccade amplitude, the values on dysconjugacy during and after the saccades are presented as the percentage of ratio of the disconjugacy on the saccade amplitude.

**Figure 5:**
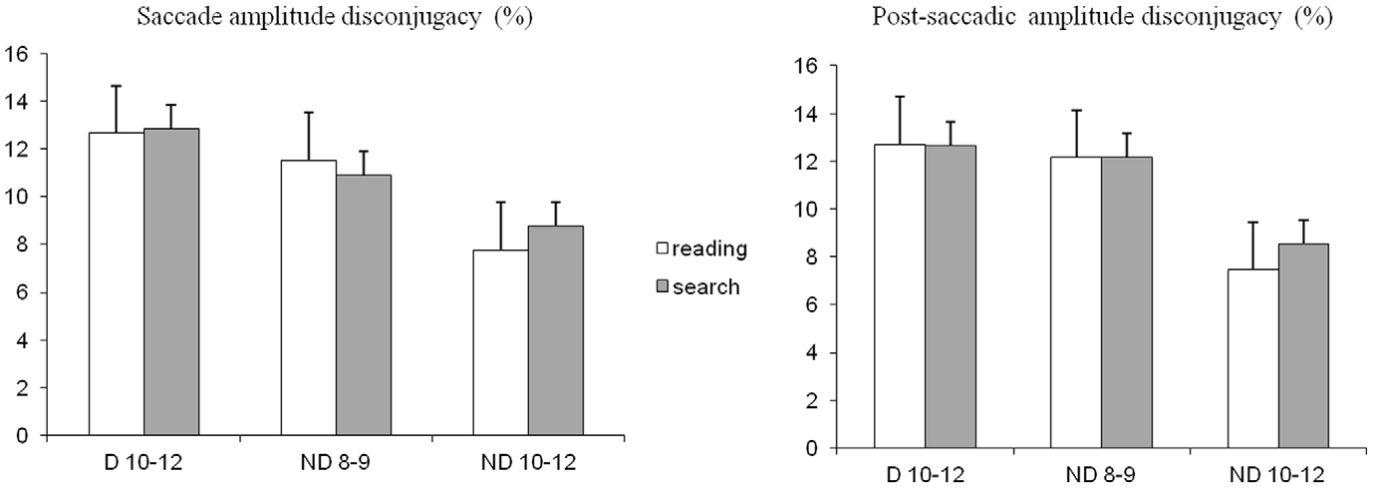
Disconjugacy during and after the saccades. Mean values of disconjugacy (measured as the percentage of ratio of the disconjugacy on the saccade amplitude) during (A) and after (B) the saccades during reading and during visual search for the three groups of children tested. Vertical lines indicate the standard error.

In Figure 5 the disconjugacy assessed during (A) and after the saccade (B) is shown. For the disconjugacy values reported during the saccade, the ANOVA showed a significant group effect (F_(2,28)_ = 19.71, p<0.00001). Post hoc comparison showed that the saccades disconjugacy of the older group of non dyslexic children was significant smaller with respect to the dyslexic group (p<0.0001) and to the younger group of non dyslexics (p<0.0001). The ANOVA did neither show a significant task effect (F_(2,28)_ = 0.24, p<0.63) nor a significant interaction between the groups of children and task (F_(2,28)_ = 0.38, p<0.69).

Similar statistical results were reported for the values of the disconjugacy measured after the saccade. The ANOVA showed a significant group effect (F_(2,28)_ = 10.16, p<0.004). Post hoc comparison showed that the disconjugacy after the saccades of the older group of non dyslexic children was significant smaller with respect to the dyslexic group (p<0.0001) and to the younger group of non dyslexics (p<0.005). The ANOVA failed to show both a significant task effect (F_(2,28)_ = 0.86, p<0.36) and a significant interaction between the groups of children (F_(2,28)_ = 1.38, p<0.27).

Finally, we have to note that we did not found out any correlation between subjective measures of vergence clinically assessed and ocular motor measures. However a previous work of Bucci et al. [25] showed that binocular coordination of saccades at reading distance in children with limited range of fusional vergence was poor and it was improved significantly after orthoptic training. Such finding suggests a relationship between saccade performance and subjective vergence capabilities (see also [32]) that, however, need to be explored further.

## Discussion

At our knowledge, it is the first time that the ocular motor behavior of dyslexic children is compared to that of reading age-matched and chronological age-matched non dyslexic children. This study shows that ocular motor characteristics of dyslexic children are impaired with respect to those reported in non dyslexic children with comparable chronological age. This finding is in agreement with many previous studies on dyslexic children. In contrast, here we reported that fixation's pattern and disconjugacy assessed during and after the saccades in dyslexic children is similar to those observed in reading age-matched non dyslexic children. Finally, another finding of this study is that for dyslexic children and for reading age-matched non dyslexic children the ocular motor behavior is similar in the two tasks, reading a text and visual search. Each of these findings is discussed.

### Pattern of fixations

Many fixations and longer duration, during reading and visual search, could be due to an immaturity of visual attentional strategies, leading to reduced visual attentional span, which corresponds to the number of elements that can be processed in parallel according to Bosse et al. [21]. Such a limitation leads to a higher number of fixations and longer fixation duration that, at least for the reading task, suggest that the child will read the text analytically. In normal children, fixation duration, during reading, decreases with age and reaches the adult level at 11 years of age [33]. On the other hand, the brain activity (frontal and parietal cortex) in children during saccade task is low relative to adults and it increases until the adolescence [34]. Furthermore, cortical structures (e.g. left temporal and parietal cortex) involved in linguistic processes are also developing with age [35], [36]. Based on all these findings, the pattern of fixation reported here in dyslexic children, as well as in reading age-matched non dyslexic children, could be related to general cortical development that is not yet completed. This hypothesis is corroborated by the pattern of fixations found in the group of chronological age-matched non dyslexic children, whose reading capabilities are well developed, and the number and the duration of fixations decreased drastically in reading task with respect to the two other groups of children.

### Disconjugacy during and after the saccades

The poor quality of binocular coordination in dyslexic children, during and after the saccades, suggests an immaturity of ocular motor learning mechanisms, at central/cortical level responsible of saccade yoking. Fioravanti et al. [37] were the first to show that saccades to LEDs in young children (<9 years old) are poorly coordinated while for older children (>11 years old), disconjugacy was smaller and similar to that observed in adults. Subsequently, Bucci & Kapoula [24] reported that in a simplified reading task (single word reading) the binocular coordination during and after the saccades in a group of 7 years old children was also significantly worse compared to that of adults. These authors suggested that the interaction between the saccade and vergence ocular motor system responsible for the execution of yoked saccades is still immature in 7 years old children. It has also been mentioned that in dyslexic children the clinically assessed limited vergence capabilities (see Table 1) could be responsible for such a deficient interaction between saccadic and vergence movements and thus lead to disconjugate saccades as those reported in the group of younger (reading age-matched) non dyslexic children. We could make the hypothesis that, as reported in children with vergence deficiencies [25] that vergence training could help dyslexic children improve the quality of their saccade coordination. This hypothesis, however, needs further exploration.

Finally with respect to magnocellular theory cited in Introduction this study does not show convincing evidences in favor of this theory; indeed our data are more in line with the hypothesis of an immaturity of learning mechanisms responsible of the fine binocular coordination of saccades. Such mechanisms could involve the magnocellular network and also the cerebellum according to the study of Nicolson et al. [38]. However, according to Iles et al. [17], deficits in the magnocellular network involving the parietal cortex could be related to poor visuo-attentional capabilities already reported in dyslexic children. Further studies by combining neuroimaging techniques and visuo-attentional tasks will be necessary to test the different hypothesis on the origin of dyslexia.

### Task effect

For dyslexic children and for reading age-matched non dyslexic children, the two tasks produced similar effects in terms of fixation as well as in terms of binocular coordination. This finding is in line with the study of Prado et al. [22] according to which a reduced visual attentional span could have a similar impact on reading and on visual search, because of the similar visual attentional demand in the two tasks. Most likely, at least for these two groups of children, for who the reading capabilities are not well structured yet, reading and visual search had similar demands in visuo-perceptual, attention and spatial processing.

In contrast, for the group of chronological age-matched non dyslexic children, the results found for the two tasks differ in the number and the duration of the fixations. Indeed, the pattern of fixation is different in the two tasks because they correspond to different cognitive demands in the case of well-reading children. For instance, in visual search task, child is required to identify and count a single target, and has to see all the letters. In contrast, in reading task, because the linguistic processing is well developed, the child can skip letters. Consequently, for these children, reading is an easier task than the visual search.

Finally, it should be pointed out that the two tasks did not show any difference regarding the binocular coordination of saccades. This is in line with previous works from Bucci [24], [26] showing that the quality of binocular coordination during and after the saccades does not depend on the stimulus used (single word reading or fixation of LEDs), and also with the study of Jainta & Kapoula [10] comparing binocular saccade coordination during reading and free exploration of painting. The present study brings new evidence on the fact that reading a text does not interfere with the quality of binocular coordination and contrasts earlier [39] and recent [12] reports suggesting that reading itself induces impairment in the binocular saccade control and fixation instability.

We should also mention that the type of the word and its font may have an influence in the vergence error recording in a reading task as shown by the study of Jainta et al. [40]. Indeed, these authors reported larger vergence error while adult subjects were fixating words with high auto-correlation (see also Wilkins et al. [41]). As suggested by these authors [40] maybe words with low auto-correlation could be easily read by dyslexic population having poor vergence control. This issue, however, need to be tested.

### Conclusion and future directions

Deficits in ocular motor behavior reported in dyslexic children seem to be due to the immaturity of their mechanism, responsible for the precise controlled interaction between the saccade and the vergence systems. Although no correlations could be found between oculomotor measures and clinical assessments, poor fusional vergence capabilities in dyslexic children may add to poor binocular coordination; this issue has to be investigated in further studies. Also, even if in the present study we did not measured the spatio-attentional capabilities of children we agree with the studies of Valdois's group [21], [22], [23] suggesting that a reduced visual attention span could be at the origin of the pattern of fixation found out in dyslexic children.

We believe that orthoptic vergence training, together with specific visual attentional training and reading tasks, could be useful tools in dyslexic children to improve visual attentional span, vergence capabilities as well as saccade yoking.
